# Outlook for CRISPR-based tuberculosis assays now in their infancy

**DOI:** 10.3389/fimmu.2023.1172035

**Published:** 2023-08-03

**Authors:** Zhen Huang, Guoliang Zhang, Christopher J. Lyon, Tony Y. Hu, Shuihua Lu

**Affiliations:** ^1^ National Clinical Research Center for Infectious Disease, Shenzhen Third People’s Hospital, Shenzhen, Guangdong, China; ^2^ Center for Cellular and Molecular Diagnostics, Tulane University School of Medicine, New Orleans, LA, United States; ^3^ Department of Biochemistry and Molecular Biology, Tulane University School of Medicine, New Orleans, LA, United States

**Keywords:** tuberculosis, diagnosis, CRISPR, point-of-care, challenge and outlook

## Abstract

Tuberculosis (TB) remains a major underdiagnosed public health threat worldwide, being responsible for more than 10 million cases and one million deaths annually. TB diagnosis has become more rapid with the development and adoption of molecular tests, but remains challenging with traditional TB diagnosis, but there has not been a critical review of this area. Here, we systematically review these approaches to assess their diagnostic potential and issues with the development and clinical evaluation of proposed CRISPR-based TB assays. Based on these observations, we propose constructive suggestions to improve sample pretreatment, method development, clinical validation, and accessibility of these assays to streamline future assay development and validation studies.

## 1 Introduction

It is estimated that one-quarter of the world population is infected with *Mycobacterium tuberculosis* (*Mtb*), and about 10 millions of these individuals develop tuberculosis (TB) annually, with more than 1 million TB-related deaths per year (1). Further, the global burden of TB and drug-resistant TB (DR-TB) has increased by 4.5% and 3% over the past year (1), deviating from the anticipated reduction rates required to meet the current schedule of the “End TB” strategy (2).

Early and accurate diagnosis of TB is critical for TB eradication efforts (3), but TB diagnosis remains challenging, and >35% of the estimated global TB cases are undiagnosed by current efforts (1, 4). This includes cases missed by insensitive sputum microbiology assays and immunoassays (5, 6), individuals who have difficulty producing diagnostic sputum samples (children, people living with HIV, extrapulmonary TB cases, or certain neurological impairments, including Dementia and Parkinson’s disease) (7–11), people living in remote high TB burden areas (4), and individuals infected with DR strain who have not undergone DR screening (1). Invasive (e.g., bronchoalveolar lavage or gastric aspirate) (12) and non-invasive (e.g., stool) (13) samples can be used as additional complementary specimens to improve pulmonary TB diagnosis in Patients that have difficulty producing expectorated sputum, while additional invasive biopsies are often required to diagnose extrapulmonary TB. However, these samples versus sputum may exhibit reduced diagnostic sensitivity and be more variable and difficult to obtain.

Research is ongoing to develop new TB biomarkers and detection technologies to enhance TB diagnosis. The application of new tools has accelerated the discovery of TB biomarkers, revealing many pathogen-derived biomarkers such as nucleic acids (*Mtb* DNA and RNA) and antigens (whole bacilli, cell components, or metabolites), as well as host-derived markers and signatures including antibodies, cytokines and chemokines, transcriptomic, proteomic and metabolic markers, and hematological effectors (14). However, only a small fraction (4%, 44/1008) of the biomarker candidates screened to date have shown diagnostic value in validation studies, and only a sputum-based PCR test for *Mtb* DNA (e.g., GeneXpert MTB/RIF, Xpert) is endorsed and promoted worldwide by the WHO for TB diagnosis (14). Xpert can rapidly diagnose TB with high sensitivity, and identify the most common form of initial drug resistance, when used to analyze sputum with high *Mtb* levels (15), but has poor diagnostic accuracy with low *Mtb* concentration (paucibacillary) samples (16) and for extrapulmonary TB (17). Its high cost also limits its accessibility in remote areas, which may explain why Xpert has not increased global TB detection rates (18). There is a pressing need for more efficient TB diagnostic tests, as described in the WHO target product profile (14, 19), which should employ easy-to-use techniques and sensitively detect or quantify TB-specific biomarkers in non-sputum samples to rapidly diagnose TB and respond to treatment.

Clustered regularly interspaced short palindromic repeats (CRISPR) sequence-specific cleavage activity provides a useful means to overcome challenges associated with TB diagnosis. CRISPR/Cas complexes utilize a short guide RNA to bind a specific target sequence, which activates their cis-cleavage activity to cut this target sequence and can also induce a trans-cleavage activity that cuts non-specific sequences while bound to its target sequence. This trans-cleavage activity can be used to repeatedly cleavage an abundant reporter oligonucleotide in proportion to the abundance of the target sequence for signal amplification (20, 21). CRISPR/Cas activity can thus be used to detect low copy number targets that differ by single-nucleotide polymorphisms (SNPs) (22, 23) to accurately detect trace nucleic acid (NA) or non-NA targets (24, 25) in complex clinical samples, including SNPs associated with microbial DR (26). CRISPR systems call also be easily integrated into portable platforms suitable for point-of-care (POC) testing (27–29). These features provide the opportunity to create CRISPR diagnostic platforms for cross-over the barriers of TB finding.

Several groups have realized the potential of CRISPR-based assays to overcome weaknesses associated with current tests employed for TB diagnosis (30–43). However, their studies largely ignore the drawbacks and challenges of CRISPR-based TB (CRISPR-TB) assays for methodology development and clinical validation studies, as these applications are still in their infancy. Here, we systematically reviewed current CRISPR-TB assays to identify their weakness, describe potential barriers to their future adoption, and propose steps that should be taken to enhance the development and translation of these assays.

## 2 Summary of current CRISPR-TB assay research

**Figure 1:**
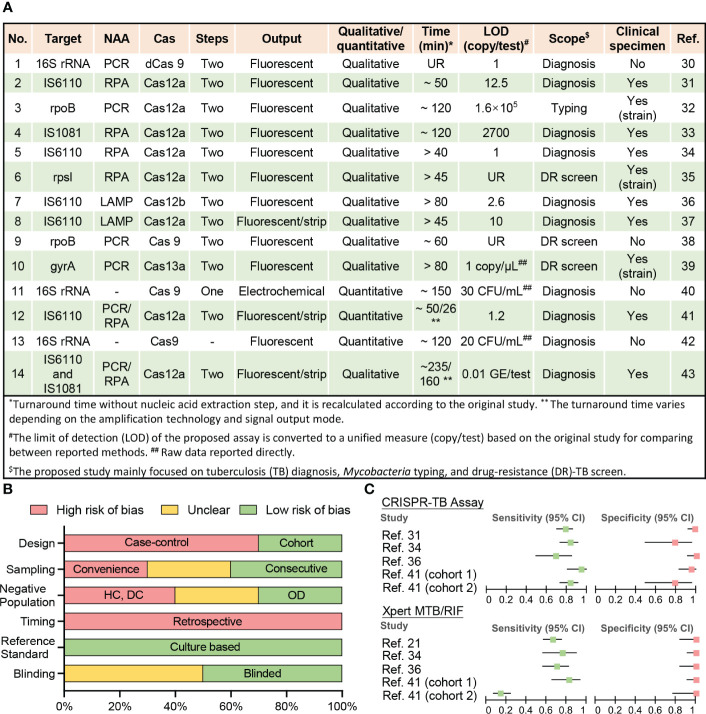
Current CRISPR-TB assay parameters and performance. **(A)** Methodological details of 12 studies employing CRISPR-TB assays. **(B)** Summary of QUADAS assessment results for the risk of bias in assessing data quality. Nine studies performed clinical sample evaluations (answers to QUADAS questions are listed in **Table S1**). The bar length reflects the frequency of an answer for each question. DC, disease contacts; DR, drug-resistant; HC, healthy control; LAMP, loop-mediated isothermal amplification; LOD, the limit of detection; NAA, nucleic acid amplification; OD, other diseases; PCR, polymerase chain reaction; RPA, recombinase polymerase amplification; UR, unreported. **(C)** Forest plot for the sensitivity and specificity of the CRISPR-TB assay and GeneXpert MTB/RIF (Xpert) results when compared with a composite reference standard. Details of the ten clinical specimen or strain validation studies, including control group information and diagnostic performance, are summarized in **Table S2**.

Fourteen studies have employed CRISPR to diagnose TB, identify DR-*Mtb* strains, and distinguish *Mtb* from nontuberculous mycobacteria (NTM) species that may produce similar symptoms but require different treatments (**Figures 1A**, **S1**). Most of these studies were published after 2019, indicating the recent nature of most of the interest in using CRISPR assays for TB diagnosis.

### 2.1 CRISPR-TB assay methodologies

Most reported assays still adhere to the original design paradigm of CRISPR assays, where CRISPR-based signal amplification is performed after an exponential nucleic acid amplification (NAA) step (21, 44, 45). This assay design detects trace levels of target NA sequences in clinical specimens to yield high analytical sensitivity (30, 34, 36, 41), but can prolong run times (> 1 h) (32, 33, 36, 39, 40, 43) (**Figure 1A**) and increase the risk of cross-contamination if amplified target NA sequences are transferred to separate CRISPR reactions. Most of these CRISPR assays employ recombinase polymerase amplification (RPA) or loop-mediated isothermal amplification (LAMP)-based isothermal amplification reactions for NAA to avoid the need for a thermocycler, which can facilitate the development of POC applications. However, many of these assays also generate fluorescent signals that require additional equipment to read and are thus less suitable for use in remote and resource-limited areas without the development of simple readout devices, although lateral flow strip-based visual readout approaches can present a good alternative for qualitative assays (37, 41, 43).

Proposed assays tend to employ *Mtb*-complex specific multi-copy genes (*IS6110* and IS1081) as diagnostic targets as it provides higher diagnostic sensitivity (31, 33, 34, 36, 37, 41, 43) and several also detects changes in the rifampicin resistance (RR)-determining region (RRDR) of the *rpoB* gene (32, 38) to screen for drug resistance, since this region is altered in 95% of RR-TB cases, and most (>78%) multi-DR TB (MDR-TB) cases (1, 46). However, other *Mtb*-complex specific sequences in single or multi-copy genes (*16s RNA*) can also be used for TB diagnosis, while DR-related mutations in other genes (*rpsl* and *gyrB*) can be employed to predict resistance to other drugs used for TB treatment. It is worth noting that the sequence conservation among *Mtb* complex species (>99%) (47) poses a challenge when attempting to distinguish individual *Mtb* complex species. CRISPR assays can have single base specificity, but many of the current TB assays use IS6110 as a target and this sequence has also been detected in all *Mtb* complex species analyzed by these assays (*M. bovis*, *M. bovis* Bacillus Calmette–Guérin, *M. africanum*, and *M. microti*) (31, 37) Further work is therefore required to identify targets that can distinguish distinct *Mtb* complex species where this information would influence treatment decisions.

### 2.2 CRISPR-TB assay study quality

Well-designed clinical studies are required to evaluate the diagnostic performance of newly developed assays but have yet to be performed for most CRISPR-TB assays (**Figure 1B** and **Table S1**). Only ten studies have analyzed clinical samples, including three studies that used clinically isolated strains instead of patient samples (**Figure 1A**). All of these seven studies exhibit high bias using a modified Quality Assessment of Diagnostic Accuracy Studies (QUADAS) evaluation (14) (**Table S1**) primarily due to their retrospective and case-control designs, lack of consecutive sampling, and the use of controls that can inflate accuracy estimates (**Figure 1B**). These studies included 1219 individuals, most of whom (74%) were from China, and a substantial fraction (41%) of these individuals lacked reported demographic information and/or clinical characteristics, preventing an accurate assessment of the potential impact of population heterogeneities and comorbidities.

### 2.3 Diagnostic performance of CRISPR-TB assays

Nine of the ten studies provided at least one microbiological test result from *Mtb* culture and Xpert or had a clear clinical diagnosis to permit accurate assessment of the diagnostic performance of the proposed CRISPR-TB assay, but only four studies (involving five cohorts) were able to provide definitive Xpert results for methodological comparisons (**Table S2** and **Figure 1C**). Unsurprisingly, CRISPR-TB assays tended to have higher diagnostic sensitivity than Xpert, with comparable or slightly decreased specificity, in most of these studies (**Figure 1C**), although these differences did not achieve significance, likely due to the limited number of individuals in these studies. However, CRISPR-TB assay sensitivity was significantly higher than Xpert (80.5% vs. 57.1%, p<0.05) for clinical TB cases with smear-negative sputum results (36). Further, CRISPR-TB assays have significant advantages over conventional tests when employed to analyze specimens that have low *Mtb* concentration (30), and thus be particularly useful in populations where this is a known problem (e.g., young children, patients living with HIV, etc.). For example, conventional TB assays exhibit very poor diagnostic performance (48, 49) in children living with HIV, representing a worst-case scenario for these assays. However, a CRISPR-based blood test for cell-free *Mtb* DNA diagnosed 83.3% of the children diagnosed with TB by microbiological finding or clinical algorithm, while Xpert sputum results identified only 14.5% of these children (**Figure 1C**) (41).

## 3 Challenges and outlook for CRISPR-TB assays

CRISPR assays are highly sensitive and specific, programmable, and easy-to-use. These features allow the ultra-sensitive detection of NA targets present at trace levels in complex samples, rapid target switching with different gRNA, and the development of streamlined assay platforms that can be operated in resource-limited settings. However, these properties, which have been extensively employed with assays for other diseases, appear to be underutilized in assays intended for TB diagnosis. As demonstrated in section-two, the methodologies of current CRISPR-TB assays are rudimentary, and high-quality clinical valuation studies are lacking.

### 3.1 Sample preparation for CRISPR analysis

Most CRISPR-TB assays employ column extraction protocols that involve multiple liquid transfers that confer a high risk for cross-contamination, and simple and efficient NA extraction procedures are not currently employed to avoid this issue. CRISPR assays are highly resistant to inhibitory components, and could, in theory, analyze specimens that have been subjected to chemical reduction or heating steps to inactivate nucleases and release target NAs from pathogens in these samples (31, 50). This would dramatically simplify sample handling and reduce contamination risks and facilitate the development of POC tests, although this approach could also reduce analytical sensitivity due since the approach would not concentrate sample NAs like conventional isolation procedures, and since inhibitory factors present in these lysates could attenuate target amplification in NAA-coupled CRISPR reactions.

Nano-/micro-technology may provide a means to balance assay sensitivity with streamlined sample processing approaches. For example, rapid procedures that enriched NAs using magnetic nanobeads (51) or fibrous materials (52, 53) can efficiently adsorb released SARS-COV-2 RNA for *in situ* target amplification without an elution step. Similarly, a microfluidics device that uses an electric field gradient to rapidly separate free SARS-COV-2 RNA (54) from other factors by isotachophoresis can significantly improve detection efficiency and diagnostic performance. However, while these approaches have been successful in SARS-COV-2 assays using sample lysates, significant optimization may be necessary to employ these methods for TB diagnosis, since *Mtb* lysis and nuclease deactivation steps may require more stringent conditions due to the structure and composition of the *Mtb* cell wall, and greater potential nuclease contributions from *Mtb* and its diagnostic clinical specimens. Further, optimized sample preparation procedures may need to be established for different specimen types (e.g., blood, urine, cerebrospinal fluid, stool, etc.) to permit their use in clinical applications. Incorporating the detection of non-sputum specimens into the scope of CRISPR diagnostics will maximize its ultra-sensitive properties and increase the microbiological confirmation rate of TB, which is a challenge for traditional NAA techniques.

CRISPR assays can also be used to sensitively detect non-NA targets using well-designed approaches where the binding of a functional NA reagent (e.g., aptamer/target NA complex) to a non-NA target releases a target NA sequence recognized by a CRISPR assay (24). CRISPR-TB assays could thus also potentially detect novel non-NA TB biomarkers in noninvasive or minimally invasive samples, such as *Mtb-*derived peptides or LAM in blood (55) or urine (56). Such approaches could provide additional opportunities to diagnose extrapulmonary TB, pediatric TB, and HIV-positive TB cases (57, 58) who are typically diagnosed with reduced sensitivity by standard methods and who are at increased risk for TB-related mortality. However, given the low and highly variable levels of valuable biomarkers in different samples, differentiated and efficient sample pre-treatment protocols may be required for different types of non-NA markers and different sample types. In addition, it may be critical to construct signal transduction systems with high matrix tolerance and compatibility with non-NA marker types to convert non-NA markers not identified by the CRISPR system into recognizable NA signals.

### 3.2 CRISPR assay workflow optimizations

Current CRISPR-TB assays typically utilize a separate NAA step to simplify assay development, but this increases the complexity, completion time, and contamination risk of the assay. The NAA and CRISPR reactions can be integrated into a single tube if an external force (e.g., centrifugation) is used to introduce CRISPR reagents after completion of the NAA step to avoid the potential for aerosol contamination during the addition of these reagents (59), although this still requires the use of consecutive NAA and CRISPR reactions that increase the sample-to-answer time of the assay.

Simultaneous NAA and CRISPR reactions can be performed in integrated NNA-CRISPR assays that use isothermal RPA or LAMP reactions for target amplification, which can simplify assay workflows to reduce assay performance times while avoiding the risk of contamination (51, 60, 61). However, such integrated NAA CRISPR reactions can also reduce detection sensitivity, since their buffer conditions may be suboptimal for both reactions and since these reactions are in direct competition (target amplification versus target cleavage), leading to assay designs that favor the NAA reaction to allow target accumulation over CRISPR cleavage and target detection.

It is also possible to eliminate the NAA step to simplify assay workflows and reduce reagent costs and the risk of cross-contamination, but it can significantly reduce assay sensitivity and thus necessitate the use of an ultrasensitive signal readout (62–65) or amplification system (66, 67) to detect weak signals produced in response to low concentration NA targets.

Thus, a CRISPR assay design must consider the workflow and diagnostic performance requirements for its intended application. For example, assays designed to have high diagnostic performance for paucibacillary TB cases (extrapulmonary TB, pediatric TB, and HIV-positive TB) may sacrifice procedure simplicity for sensitivity, while an assay intended as a POC test for TB diagnosis in the general population may prioritize a streamlined workflow over ultrasensitive detection.

### 3.3 Multiplex assays, target constraints, and quantitative assay readouts

Current CRISPR assays typically detect a single target and are non-quantitative. However, assays that detect a single target may produce false negatives due to strain-specific sequence variations. For example, a test that targets the multi-copy IS6110 insertion element should produce false negative results for *Mtb* strains that lack this insertion element (68, 69). Similarly, single target assays for DR-TB may miss alternate mutations in a gene associated with drug resistance and cannot detect mutations in other genes that confer resistance to other important drugs employed in anti-TB treatment regimens. For example, a test for DRTBRB targeting the RRDR fragment of rpoB to detect major mutations associated with rapamycin and rifabutin resistance could miss MDR-TB cases that lack these mutations (70).

Multiplex CRISPR assays could help address these issues, but are technically challenging to develop as single reaction tests since the trans cleavage activities of the CRISPR/Cas variants used for signal readout in the most popular and sensitive assays lack strong sequence specificity. Single-reaction multiplex CRISPR assays that employ multiple Cas proteins with distinct trans-cleavage substrate preferences have been proposed to address this issue, but no more than four targets can be detected in one reaction due to the limited number of Cas proteins with differential cleavage preferences (22), and even among these proteins there is the potential for significant off-target cleavage and the need to optimize an assay for all four activities.

Microfluidic- or micro-droplet-based approaches may represent a better option for multiplex CRISPR assays as they can perform large numbers of distinct single-target tests in spatially separated regions to avoid target/reporter crosstalk difficulties while simultaneously detecting hundreds or thousands of distinct NA targets (71, 72).

Sequence considerations can also influence which specific target regions can be analyzed in a CRISPR assay, which can present a challenge when an assay must detect a specific sequence associated with a phenotype type of interest (e.g., a SNP associated with resistance to a specific drug). Most CRISPR/Cas systems used for sensitive NA detection require that their target NAs contain a protospacer adjacent motif (PAM) sequence, which is problematic when a sequence of interest does not contain this motif. This issue can be partially addressed by using NAA primers to introduce a PAM sequence into amplicons that contain the sequence of interest (61). However, there are limitations to this approach as this PAM sequence must be introduced in close proximity to the sequence of interest with minimal primer mismatch, and some PAM optimization may be required to obtain specificity for a SNP of interest. An alternate solution is to screen for or bioengineer Cas protein variants that exhibit fewer PAM constraints (73). Cas14 can recognize and cleave single-stranded DNA targets that lack PAM sequences (74), and could serve as a template for the design of new CAS proteins that lack a PAM sequence requirement.

Quantitative CRISPR assays are also necessary to rapidly determine *Mtb* burden and its real-time response to anti-TB therapy as a measure of disease severity and treatment efficacy (41). Standard curves can be used to quantify *Mtb* DNA levels in clinical specimens but can produce highly variable results when analyzing samples that contain only trace amounts of a target sequence (41). CRISPR assays that employ digital droplet technology to achieve absolute quantification can circumvent this problem (23, 75–80), but this approach requires additional equipment and resources. Smartphones have the signal acquisition and data processing properties required for portable quantitative assays suitable for use in resource-limited areas, and their network connectivity also provides a convenient means for data reporting for disease control efforts. Thus, the combination of CRISPR-based TB assays and smartphone-based readout devices, or other similar portable devices, has the potential to increase the capacity for TB screening and treatment monitoring.

### 3.4 Clinical validation studies

CRISPR-TB assays have exciting potential to improve TB diagnosis and management, but their translation as clinical applications require their validation in well-designed, adequately powered, and multicenter prospective clinical studies, which have not been conducted for any of the CRISPR-TB assays that have been reported to date. Such studies should ideally include cohorts of extrapulmonary TB, pediatric TB, and HIV-positive TB cases, as these individuals would most benefit from early diagnosis and treatment initiation to reduce their high mortality rates. These studies should also evaluate the relative utility of CRISPR-TB assay results obtained from several types of noninvasive or minimally invasive patient specimens (e.g., urine, stool, fingerstick blood samples) for TB diagnosis in different populations, and for their potential application as CRISPR-TB assays intended for use in resource-limited settings where obtaining sputum or invasive specimens can be difficult or infeasible. Moreover, given the high sensitivity CRISPR, it is recommended that these clinical evaluation studies employ a composite criterion to identify the TB-positive and TB-negative individuals, since the use of a single standard diagnostic method may misdiagnose TB cases, particularly in extrapulmonary, pediatric, or HIV-positive cohorts to skew CRISPR-TB assay sensitivity and specificity estimates.

### 3.5 Assay accessibility

Future studies should also focus on improving the accessibility of CRISPR-TB assays, as limited clinical laboratory resources or infrastructure may reduce access to or capacity for TB diagnostic tests in areas with high TB incidence and prevalence rates. Such assays should ideally integrate a rapid NA extraction method into the CRISPR-TB assay and employ a rapid and streamlined procedure that does not require significant additional equipment to perform. Ideally, such assays would integrate all their procedures into a single streamlined assay platform (e.g., a microfluidic chip or a test strip) in a direct sample-to-result assay format that would not require technical expertise or any equipment for sample processing or assay readout. Such integrated platforms could potentially be into wearable devices, such as masks (27), for streamlined real-time assessment TB assessment (81). Negative and positive controls should be incorporated into these platforms to permit immediate evaluation for adverse storage and contamination effects and other confounding factors that could decease the accuracy of assay results.

CRISPR-TB assays intended for use in remote and resource-limited areas should also account for the transport and storage conditions these assays will likely face, ideally during their initial development phase, since cold chains are often difficult to maintain in areas with high TB burden. Lyophilized CRISPR reagents can be stored for months at four degrees and weeks at room temperature without significant performance decreases (28, 82). However, assay developers should also determine the stability of a CRISPR-TB assay at the more variable ambient temperatures these assays might be likely to encounter in areas without temperature control. The efforts will also be required to promote the large-scale production of key reagents, such as Cas proteins, to reduce assay development and production costs and to shorten distribution distances, which will require a streamlined licensing procedure for the relevant patents, as has been done for NAA reagents.

## 4 Perspective on new CRISPR-TB assay development

**Figure 2:**
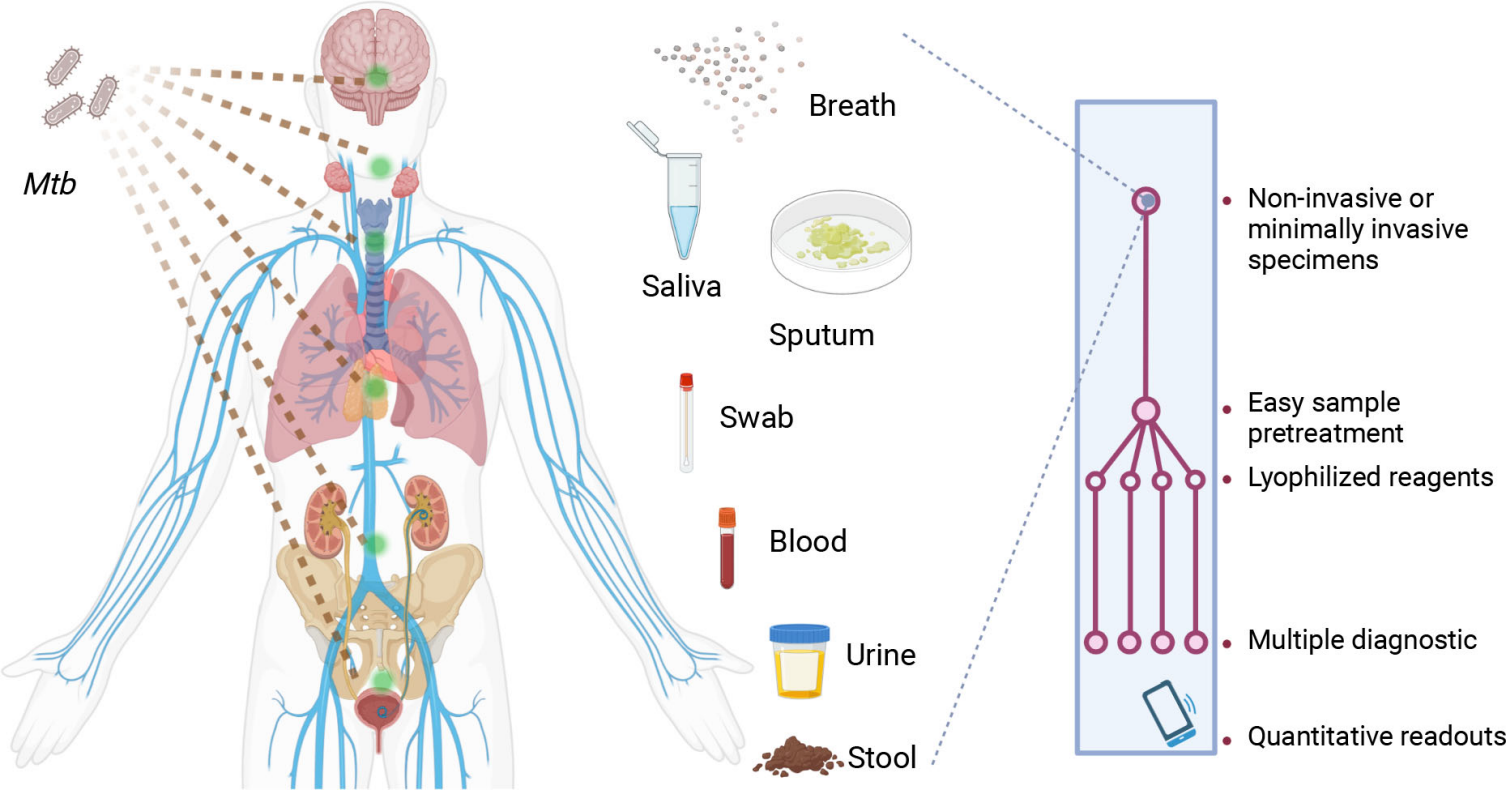
Characteristics desired for future CRISPR-TB assays. Future CRISPR-TB assays should ideally analyze noninvasive or minimally invasive diagnostic specimens (e.g., breath aerosol, saliva, sputum, swab, blood, urine and stool samples) and employ an integrated platform with lyophilized reagents to minimize cold chain concerns. These assays should integrate sample treatment and target detection reactions in a streamlined workflow to provide assay results within minutes. The assay readout should also provide quantitative results when read by a smart terminal, and this device should be able to report these results to a central system to facilitate TB control efforts or telemedicine interventions. *Mtb*, *Mycobacterium tuberculosis*.

In summary, CRISPR-TB assays have strong potential to improve the TB diagnosis of TB, but clinical validation studies are required to allow regulatory approval and commercialization for TB diagnosis and treatment evaluation. Substantial refinements are usually also required to translate an initial proof-of-concept CRISPR-TB assay suitable for use in a research laboratory to a clinical application that can be employed at a large-scale in clinical laboratories, clinics, or POC settings. We propose that future CRISPR-TB assays (**Figure 2**) should ideally employ an integrated platform for sample processing, NA enrichment, and coupled NAA and CRISPR detection that contains lyophilized reagents to minimize assay cold chain concerns. Such platforms should evaluate noninvasive or minimally invasive diagnostic specimens, employ streamlined workflows with rapid sample-to-result times, and employ a readout that can be quantified by a smart terminal that can report results to a central system to aid in TB control efforts or telemedicine interventions. We believe maturing CRISPR-TB assay approaches represent a powerful means of addressing current TB diagnosis and treatment evaluation challenges required to achieve the goals of current TB eradication efforts. Mature CRISPR-TB assays may also prove valuable in non-clinical applications, such as screening for active *Mtb* or *Mtb* complex infections in domestic livestock or wildlife populations (83, 84) as has been done with Xpert. This would be particularly valuable is these analyses could employ blood or fecal specimens to simplify sample collection or population level screening efforts.

## Author contributions

ZH and SL conceived the design of this manuscript. ZH and GZ performed the dataset search, article screen, data extraction, and quality appraisal. ZH drafted the manuscript, and GZ, CL, and TH provided critical revision. All authors contributed to the article and approved the submitted version. SL was responsible for the decision to submit the manuscript.
