# Supplementary material for: Outlook for CRISPR-based tuberculosis assays now in their infancy
Source: Front Immunol. 2023 Aug 3;14:1172035. doi: 10.3389/fimmu.2023.1172035 (PMC10436990; doi:10.3389/fimmu.2023.1172035)
Supplement: Supplementary file 1 [file DataSheet_1.docx]

**Supporting Information for**

**Outlook for CRISPR-based tuberculosis assays now in their infancy**

Zhen Huang^1*^, Guoliang Zhang^1^, Christopher J. Lyon^2,3^, Tony Y. Hu^2,3^, Shuihua Lu^1*^

^1^ National Clinical Research Center for Infectious Disease, Shenzhen Third People’s Hospital, Shenzhen, Guangdong Province 518112, China.

^2^ Center for Cellular and Molecular Diagnostics, Tulane University School of Medicine, New Orleans, LA 70112, USA.

^3^ Department of Biochemistry and Molecular Biology, Tulane University School of Medicine, New Orleans, LA 70112, USA.

Corresponding to Dr. Zhen Huang and Prof. Shuihua Lu*:

Address: No.29 Bulan Road, Shenzhen, Guangdong 518115, China.

Dr. Zhen Huang: Tel: +1-304-506-7992, Email: huang.zhen@outlook.com

Prof. Shuihua Lu: Tel: +86-189-3081-1818, Email: lushuihua66@126.com

**Fig. S1**


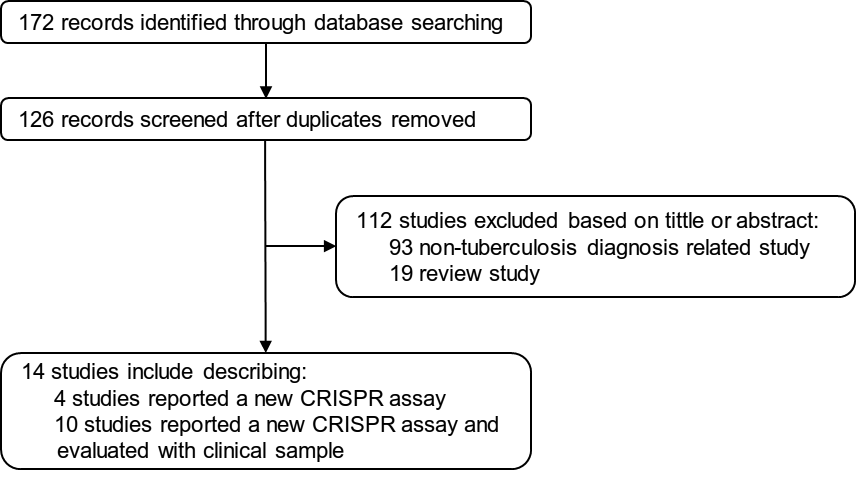


**Supplementary Figure 1. Flow chart of publication selection.** The keywords of "CRISPR", "Tuberculosis", and "Diagnosis" or their synonyms were used to search without language limitation in the major academic databases, including PubMed, EMBASE, Web of Science, and Google scholar. After deduplication, 124 publications were identified, with 112 excluded at the title/abstract screening stage as they were not eligible. The left 12 full-text articles met the criteria for final inclusion.

| **Supplementary Table 1. QUADAS quality evaluation results of involved ten studies*** | | | | | | | | | | |
| --- | --- | --- | --- | --- | --- | --- | --- | --- | --- | --- |
| **Ref.** | **31** | **33** | **34** | **36** | **37** | **41** | **32** | **35** | **39** | **43** |
| **Design** (Domain "Patient selection) | | | | | | | | | | |
| Q: Was a case-control design avoided? | Y | N | N | Y | N | Y | N | N | N | N |
| **Sampling** (Domain “Patient selection”) | | | | | | | | | | |
| Q: Was convenience sampling avoided? | Y | Y | N | U | U | Y | N | N | U | N |
| **Negative population** (Domain “Patient selection”) | | | | | | | | | | |
| Q: Was the patient selection done in such a way as to avoid introduction of bias? | Y | U | U | Y | N | Y | N | N | N | U |
| **Timing** (Domain “Flow and timing”) | | | | | | | | | | |
| Q: Was the study prospective in timing? | N | N | N | N | N | N | N | N | N | N |
| **Reference standard** (Domain “Reference standard”) | | | | | | | | | | |
| Q: Did the reference standard or composite reference standard include culture? | Y | Y | Y | Y | Y | Y | Y | Y | Y | Y |
| **Blinding** (Domain “Index test”) | | | | | | | | | | |
| Q: Was the index test interpreted in a blinded manner? | U | Y | U | U | Y | Y | Y | U | Y | U |
| * The QUADAS quality evaluation protocol adapts form **Ref. (Nat. Microbiol., 2019, 4: 748–758)** Y= yes, U= unreported, N=no | | | | | | | | | | |

| **Supplementary Table 2. Diagnosis performance evaluation of the proposed CRISPR-based tuberculosis assays** | | | | | | | | | | |
| --- | --- | --- | --- | --- | --- | --- | --- | --- | --- | --- |
| **No** | **Ref.** | **Species type** | **TB cases (n)** | **Control (n)** | **CRISPR-TB assay** | | **Xpert** | | **Culture** | |
|  |  |  |  |  | **Sensitivity**  **(%, 95% CI)** | **Specificity**  **(%, 95% CI)** | **Sensitivity**  **(%, 95% CI)** | **Specificity**  **(%, 95% CI)** | **Sensitivity**  **(%, 95% CI)** | **Specificity**  **(%, 95% CI)** |
| 1 | 31 | Sputum, CSF, pus, urine, and serous cavity fluid | 116  (85 microbiological confirmed TB and 31 clinical diagnosed TB) | 63  non-TB (other diseases) | 78.5  (69.9-85.5) | 98.4  (91.5-100.0) | 66.38  (57.0-74.9) | 100  (94.3-100.0) | 32.8  (24.3-42.1) | 100  (94.3-100.0) |
| 2 | 33 | Sputum | 140  (*Mtb* culture positive) | 54  (*Mtb* culture negative) | 99.3  (96.1-100) | 100.0  (93.3-100.0) | NA | NA | Ref | Ref |
| 3 | 34 | Sputum | 29  (*Mtb* culture or Xpert positive cases) | 40  (*Mtb* culture and Xpert negative cases) | 69.0  (49.2-84.7) | 100.0  (91.2-100.0) | 75.9  (56.5-89.7) | 100.0  (91.2-100.0) | 86.2  (68.3-96.1) | 100.0  (91.2-100.0) |
| 4 | 36 | Sputum and BALF | 68  (Microbiological confirmed TB) | 21  (Other diseases) | 86.8  (76.4-93.8) | 95.2  (76.2-99.9) | 70.4  (56.4-82.0) | 100.0  (83.9-100.0) | 66.7  (53.7-78.1) | 100.0  (83.9-100) |
|  |  | Plasma | 128  (Microbiological confirmed TB and clinical diagnosed TB) | 42  (20 cases with other diseases and 22 T-spot negative contacts) | 36.7  (28.4-45.7) | 88.1  (74.4-96.0) | NA | NA | NA | NA |
| 5 | 37^#^ | Sputum | - | - | - | - | - | - | - | - |
| 6 | 41 | Plasma (Cohort 1) | 34  (30 microbiological confirmed TB and 4 clinical diagnosed TB) ^†^ | 39  (non-TB household contacts) | 94.1  (80.3-99.3) | 94.9  (82.7-99.4) | 82.4  (65.5-93.2) | 100.0  (91.0-100.0) | 73.9  (51.6-89.8) | 100.0  (91.0-100.0) |
|  |  | Plasma (Cohort 2) | 78  (13 confirmed TB and 75 unconfirmed TB) ^†^ | 14  (Unlikely TB children without any NIH TB symptoms) ^†^ | 83.3  (73.2-90.82) | 78.6  (49.2-95.3) | 16.2  (8.7-26.6) | 100.0  (76.8-100.0) | 11.5  (5.4-20.8) | 100.0  (76.8-100.0) |
| 7 | 33^$^ | Plasma  (South African) | 11  (cfDNA-positive) | 0 | 91 | - | NA | NA | NA | NA |
|  |  | Plasma (Uganda) | 9  (*Mtb* culture or Xpert positive) | 26  (Healthy control) | 89 | 100 | NA | NA | NA | NA |
| 8 | 32 | Strains isolated from sputum | 10  (*Mtb* strains) | 63  (NTM strains) | 100.0  (69.2-100.0) | 100.0  (94.3-100.0) | NA | NA | Ref | Ref |
| 9 | 35 | Strains isolated from sputum | 49  (STR resistant *Mtb* strains confirmed by sequencing) | 40  (non-STR resistant *Mtb* strains confirmed by sequencing) | 100.0  (92.8-100.0) | 100.0  (91.2-100.0) | NA | NA | NA | NA |
| 10 | 39 | Strains isolated from sputum | 35  (Fluoroquinolone resistance *Mtb* strains confirmed by drug-susceptibility test) | 40  (non-fluoroquinolone resistance *Mtb* strains confirmed by drug-susceptibility test) | 91.4  (76.9-98.2) | 100.0  (91.2-100.0) | NA | NA | Ref | Ref |
| ^#^ Any meaningful clinical information was not present in the paper for estimates the diagnostic performance.  ^†^ The children involved in this study were categorized by NIH paediatric tuberculosis case definitions.  ^$^ The study included a limited number of individuals, so the 95% confidence interval of sensitivity and specificity of the assay was not estimated, and the clinical information is not disclosed for presenting the diagnostic information of Xpert and culture.  Mtb = Mycobacterium tuberculosis; CSF = cerebrospinal fluid; BALF = bronchoalveolar lavage fluid; STR = streptomycin-resistant; NA = not application; Ref = reference. | | | | | | | | | | |
